# Microstructure and Mechanical Properties of Solid-State Rotary Friction Welded Inconel 713C and 32CrMo4 Steel Joints Used in a Turbocharger Rotor

**DOI:** 10.3390/ma16062273

**Published:** 2023-03-12

**Authors:** Krzysztof Szwajka, Joanna Zielińska-Szwajka, Tomasz Trzepieciński

**Affiliations:** 1 Department of Integrated Design and Tribology Systems, Faculty of Mechanics and Technology, Rzeszow University of Technology, ul. Kwiatkowskiego 4, 37-450 Stalowa Wola, Poland; kszwajka@prz.edu.pl; 2 Department of Component Manufacturing and Production Organization, Faculty of Mechanics and Technology, Rzeszow University of Technology, ul. Kwiatkowskiego 4, 37-450 Stalowa Wola, Poland; j.zielinska@prz.edu.pl; 3 Department of Manufacturing Processes and Production Engineering, Rzeszow University of Technology, al. Powstańców Warszawy 8, 35-959 Rzeszów, Poland

**Keywords:** Inconel 713C, hardness, rotary welding, welded joints, welding

## Abstract

The aim of this work was to determine the effect of selected parameters of friction welding, such as friction pressure and welding speed, on the mechanical properties and microstructure of friction-welded Inconel 713C-32CrMo4 joints. Tensile strength and hardness tests were carried out to determine the mechanical properties of the resulting welded joints. The results of the ultimate tensile strength, hardness, and microstructure were linked to the parameters of the welding process. It was found that the highest tensile strength was 1222 N/mm^2^. There was a significant increase in the hardness value in the thermo-mechanically affected zone for all samples. However, as the friction pressure increased, the zone with the higher hardness value migrated towards the 32CrMo4 material. In all weld tests, the fracture was found on the 32CrMo4 steel side. A distinct band of carbide formation was observed between the thermo-mechanically affected zone and the Inconel 713C base material.

## 1. Introduction

Conventional fusion welding, as well as welding with a laser or electron beam, has a very limited application due to the melting zone of the joined materials. In conventional friction stir welding, the cylindrical tool is rotated and immersed in the weld area between two materials [1,2]. After cooling down, the joint has a small heat affected zone and similar mechanical and physical properties to the base metal (BM) [3]. The parameters of the welding process (welding time, tool rotational speed, pressure force) should be selected depending on the type and dimensions of the welded elements [4]. These welding parameters affect the depth and speed of the heating of the layers of the joined materials and the width of the heat affected zone.

By welding, materials can be joined to form linear [5,6], point [7], butt [8], circumferential [5] or eccentric [9] welds. The friction welding process consists of the deliberate use of the conversion of kinetic energy, generated as a result of friction, into thermal energy. Friction stir welding does not require any auxiliary substances, fluxes, or shielding gases. Friction welding allows for a permanent joint of elements made of various similar or dissimilar materials, such as aluminium, titanium, copper and their alloys, carbon steel, tool steel, stainless steel, and nickel alloy [10,11]. Friction welding is widely used in the automotive industry, as well as in the aviation industry and by manufacturers of cutting tools, shafts, and pipes [12,13].

Rotary Friction Welding (RFW) is a solid-state welding process where two or more parts are axially pressed onto each other under high relative rotational movement. The RFW method has been used for numerous applications, including drill pipes, piston rods, turbine discs, turbines for aircraft engines, steel truck axles, heat exchangers, and passenger car wheel rims [14,15,16]. The most commonly studied parameters of the rotary friction welding are temperature, axial force and pressure, rotation speed, and burn-off length [17,18,19,20].

The process of conventional rotary friction welding consists of joining two elements by bonding in the solid state, one of which is placed in a stationary holder and the other is subjected to rotational movement relative to the common axis. The elements get closer to each other and are pressed against each other with a certain force. Work is generated on the contact surface to overcome friction, which is then converted into heat. The surfaces of the elements heat up to a high temperature, close to the melting point of the joined metals, and are then pressed together by forge pressure to create a permanent joint. The rubbing action can be achieved by a rotary or a linear motion, or a combination of the two—called orbital friction welding [21]. In inertia friction welding (IFW), the rotating part is connected to a flywheel, which is disconnected from the drive motor once a desired rotating speed is achieved. In direct driver RFW, the rotating part is continuously driven by the equipment’s spindle motor. During the IFW process, which is a variety of the RFW process, one part is fixed on a rotating flywheel and makes contact with another part that is stationary [22]. After frictional heat and the high-temperature thermoplastic state of the materials to be joined has been achieved, a pressure force is applied to join the two parts. In an IFWed joint, there is no obvious difference between the edge and the centre region of the weld [23]. Moreover, because the material is not melted in this process, in contrast to fusion welding methods, inertia friction welding can avoid defects such as lack of penetration, poor fusion, and solidification cracks [24,25]. The other advantages are a narrow heat-affected zone (HAZ) [26], local heating [27], and a short operating time [28].

In previous works, Skowrońska et al. [29] found that the character of the weld in a dissimilar joint is based on deep plastic deformation without visible diffusive processes in the interface zone. Xie et al. [30] analysed the effects of welding parameters on microstructure, tensile strength, and microhardness in RFWed joints produced without upset forging on pure molybdenium rods. The molybdenium joints had asymmetric, spiral shaped flashes. However, the RFW of common metals produced an axially symmetrical flash. Ni-based superalloys are a wide group of materials subjected to friction welding. There are two broad categories of nickel alloys: precipitation-hardened alloys and solid-solution-strengthened alloys [31,32]. Nickel alloys in both categories can be welded using common processes [33]. Rehman et al. [34] investigated the mechanical properties and microstructure of Inconel 718-F22 RFW joints. The results revealed that the higher hardness was observed at the weld interface of the Inconel 718/2.5Cr-1Mo (F22) steel RFWed joints compared with the RFWed joints with an Inconel 625 interlayer. Firmanto et al. [35] used the RFW technique to investigate the characteristics of 304 stainless steel and low-carbon steel joints. Three factors, namely friction time, friction pressure, and upset pressure, had a significant influence on the tensile strength. Rehman et al. [36] analysed the room temperature tensile properties and microstructural characteristics of Inconel 600/Inconel 718 RFW joints. The welds were also free from Laves-phase formations and elemental segregation, which is in agreement with the results of Damodaram et al. [37] and Kong et al. [38]. The microstructure in the thermo mechanically affected zone (TMAZ), weld interface (WI) and heat-affected zone of RFWed carbon steel (EN24)/Inconel (718) were examined by Gaikwad et al. [39]. They concluded that hardness variation across the weld joint is attributed to strengthening precipitates, phase transformation, and grain refinement. Demouche et al. [40] studied the effect of RFW parameters on the microstructure of 100Cr6 steel. The results revealed that the tensile strength of RFWed samples increases with increasing welding time. Hardness was lower at the periphery of the weld joint in comparison with its centre. Madhusudhan et al. [41] have applied the RFW method to join MDN-250 maraging steel to EN-24 steel with an interlayer of nickel. It was found that nickel as an interlayer can be employed as an effective barrier to the diffusion of chemical elements. Wang et al. [42] investigated the microstructure evolution of 304 stainless steel joined by RFW. They found that the dynamic recrystallisation mainly occurs in the WI and is completed in the torque quasi-steady stage. Taysom et al. [43] found there is no correlation between the upset symmetry of the weld of dissimilar nickel-based superalloys and the weld strength that was consistent between the multiple alloy/geometry systems. Cheepu et al. [44] analysed the tensile strength, joint temperature, interface microstructure, and microhardness of friction-welded AISI304 AISI 4340 steels. They found that the temperature of the joint interface depends on the axial shortening of the substrates at the mating surfaces and the thickness of the intermixing zone. Cheepu et al. [45] investigated the interface properties of dissimilar stainless steel and titanium welds to characterise the effect of intermetallic compounds on the tensile strength of the joints. They found that the increase in heating time and upsetting pressure caused an increase in the tensile strength of the welded joints. Cheepu and Susila [46] studied the growth rate of intermetallics in the dissimilar welding of copper to aluminium. Cheepu and Che [47] found that the increase in friction pressure causes a progressive increase in the axial shortening. Cheepu and Susila [48] analysed the joining of dissimilar materials (titanium and stainless steel) to develop friction-welded joints. They concluded that intermixing regions and the work hardening phenomenon directly influence the hardness in the weld zone. Abhijith and Reddy [49] used RFW to weld the dissimilar materials of SS300 stainless steel and Inconel 600 and found that the frictional pressure would only influence penetration at the interfaces between the joined materials. It would be interesting to study the effect of the welding parameters on the microstructure and mechanical properties of the RFWed joint of 32CrMo4 constructional steel to Inconel 713C. To the authors’ best knowledge, no reports on the rotary friction welding of these materials have been published, and such a study is needed to understand the role of friction welding parameters on the strength of the joint. This study shows the analysis of the mechanical properties and microstructure of the Inconel 713C-32CrMo4 friction-welded joint.

## 2. Materials and Methods

### 2.1. Materials

**Figure 1:**
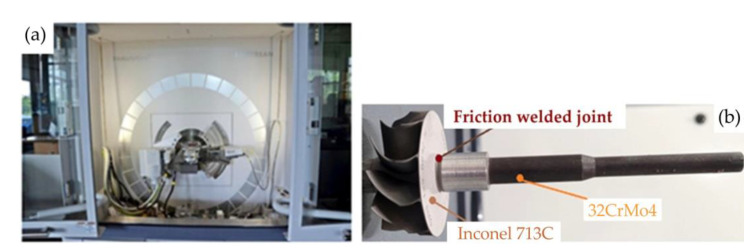
(**a**) XRD diffractometer and (**b**) turbocharger rotor with shaft.

**Figure 2:**
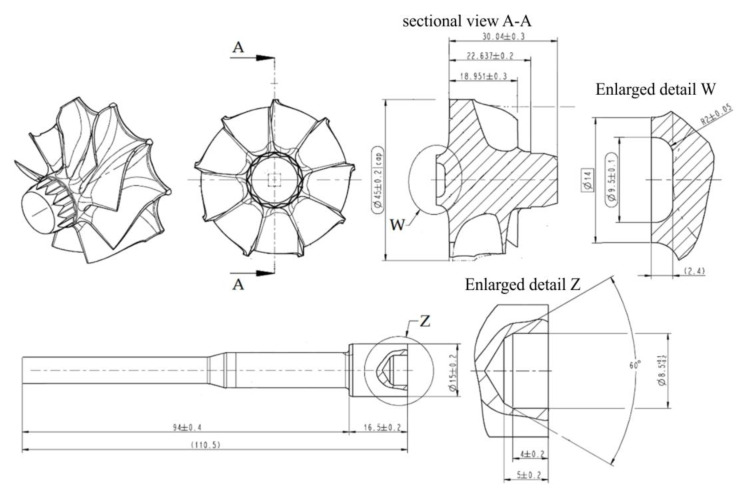
Selected dimensions of the elements joined in the RFW process.

RFW was performed on shafts made of 32CrMo4 constructional steel and rotors made of Inconel 713C material. Inconel 713C is a precipitation-hardenable, nickel-chromium base cast alloy. The elements subjected to the friction welding process form the turbocharger turbine rotor (Figure 1b). A drawing showing the important dimensions of the elements joined in the RFW process is shown in Figure 2. The outer diameter of the shaft was 15 ± 0.2 mm, and the outer diameter of the rotor was 14 mm. The chemical compositions of the welded materials were determined using X-ray diffraction (XRD) analysis on an Empyrean diffractometer (Malvern Panalytical Ltd., Malvern, UK). The chemical compositions of 32CrMo4 steel and Inconel 713C are presented in Table 1 and Table 2, respectively.

**Figure 3:**
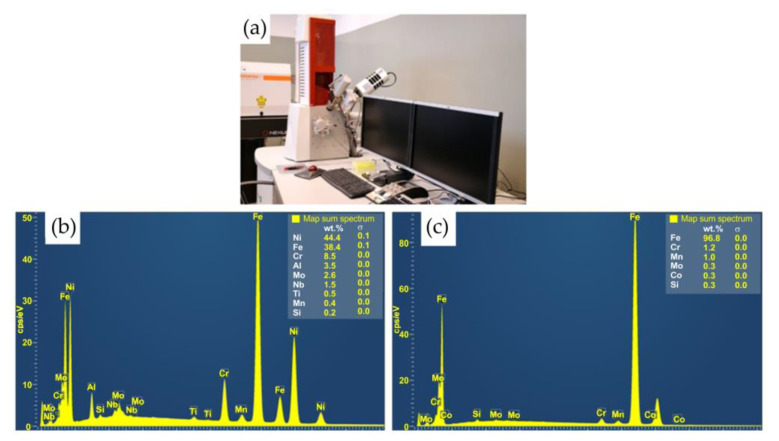
Scanning electron microscope and spectral analysis of the materials: (**a**) measuring stand; (**b**) spectra of the Inconel 713C and (**c**) spectra of the 32CrMo4 steel.

The spectral analysis of the elements constituting the material (Figure 3) was carried out using a TESCAN^®^ MIRA3 scanning electron microscope (Brno, Czech Republic).

### 2.2. Welding Procedure

**Figure 4:**
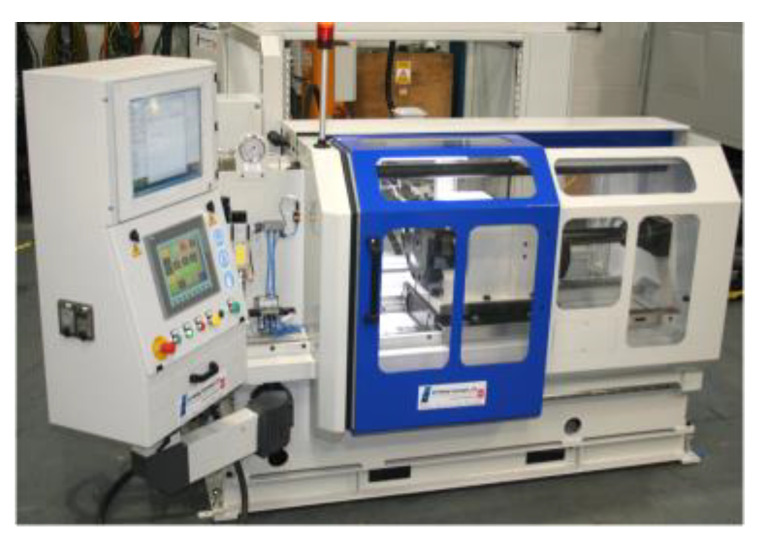
Friction welding machine MTI150B.

Friction welding of the turbocharger rotor was performed using a MTI150B friction welding machine from MTI Manufacturing Technology, Inc. (South Bend, IN, USA) (Figure 4). The machine consists of several main components, including a precision control system equipped with a recorder of the welding cycle parameters and a high-speed spindle with a pneumatic piston to generate pressure during the friction and upsetting phases. The machine includes a high-speed spindle. The friction pressure is applied by means of a pneumatic piston.

The shaft (32CrMo4 steel) is mounted in the spindle and rotated about the common axis with the fixed turbocharger wheel (Inconel 713C) mounted in a machine vice. Table 3 shows the set of friction welding parameters used during the tests. The range of welding parameter changes was selected to ensure the shortest welding time. All experiments were repeated three times. In total, 18 friction welding trials were carried out. Three repetitions were made for each welding parameter (Table 3). One set of tests was used to analyse the microstructure and measure the microhardness of the joint. Welded joints obtained in two successive repetitions were used to test the strength of the joint in a static tensile test.

**Figure 5:**
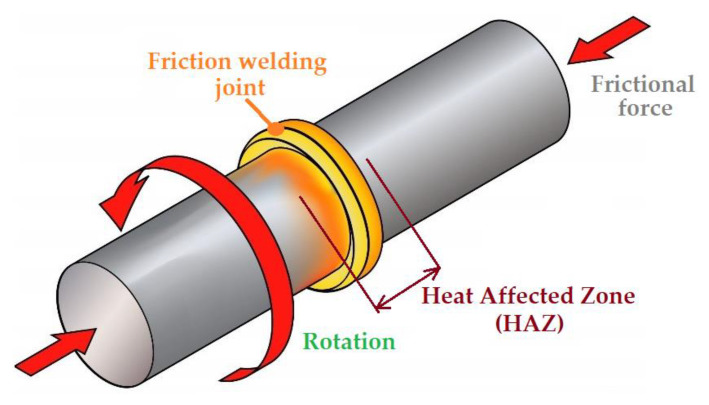
Scheme of the RFW process.

Conventional RFW is divided into two basic phases: friction and upsetting. During the first phase, heat is released at the border of the welded elements during mutual friction. In the second phase, known as the upsetting phase, the heated front surfaces of both elements connect and cool each other, creating a permanent joint. The effect of the technology used here depends on the correct selection of the process parameters for welding. These parameters include rotational speed, friction pressure, friction time, upsetting force, and upsetting time. The rotational speed determines the rate of heating in the contact area; the friction pressure affects the rate of heating in the HAZ (Figure 5); the time of friction determines the correct heating in the weld zone area; and the upsetting force and time determine the quality of the metallic joint of the surfaces heated to the plastic state. Figure 5 shows a simplified diagram of the welding process with parameters that changed during the tests. In addition, the heat affected zone in the welding process is marked.

In the tests, rotational speed and friction pressure were assumed as variable parameters due to the fact that they mainly determined the rate of heating of the joined materials. This, in turn, makes it possible to shorten the duration of the friction welding process. One of the main objectives of the research was to evaluate the economic aspects of the joints. At present, research is being carried out by the authors, in which the remaining parameters of friction welding are introduced to the experiments.

**Figure 6:**
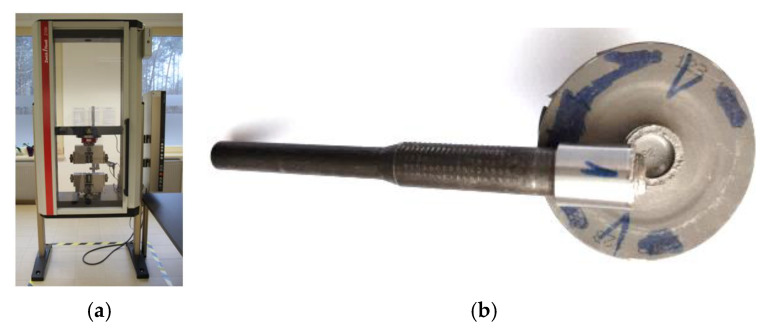
(**a**) uniaxial tensile testing stand (Zwick/Roell Z100) and (**b**) the turbine rotor after the tensile test.

The static tensile test of the welded joints were carried out on a Zwick/Roell Z100 machine (Zwick Roell Group, Ulm, Germany). Figure 6a,b shows the test stand for testing the tensile strength of the friction-welded elements and the turbine rotor, respectively.

**Figure 7:**
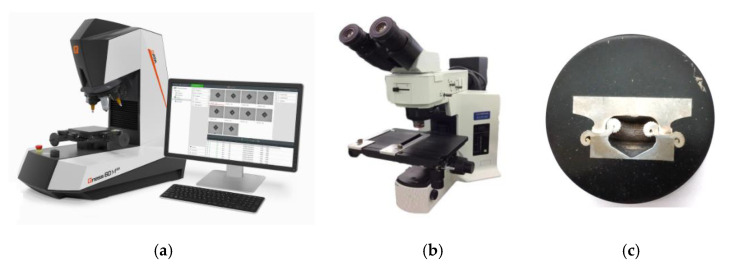
(**a**) hardness tester (Qness 60M); (**b**) optical microscope (Olympus BX51M); (**c**) the positioned specimen.

The microstructure of the friction-welded joint was characterised using an optical microscope and a scanning electron microscope (SEM) on the cross-section of the joints (perpendicularly to the rod axis) (Figure 7c). An Olympus BX51M optical microscope (Olympus Corporation, Tokyo, Japan; Figure 7b) was coupled with a digital camera with magnifications from 50× to 1000×.

**Figure 8:**
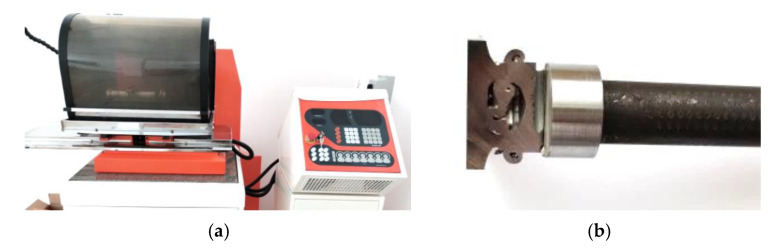
(**a**) BP95d electro-erosion cutter; (**b**) the place from which the samples were cut from the friction-welded rotor.

SEM analyses were performed on a TESCAN^®^ MIRA3 scanning electron microscope (TESCAN, Brno, Czech Republic) combined with energy dispersive spectroscopy (EDS) capability (Figure 2a). Measurements of the Vickers’ hardness in three characteristic areas was carried out using an Qness 60M (Qness, Mammelzen, Germany) hardness tester (Figure 7a) with a load of 500 g. The load was applied for 15 s. The samples for metallographic testing were cut using a BP95d electro-erosion cutter (Zakład Automatyki Przemysłowej B.P., Końskie, Poland) (Figure 8a). The location from which the samples were cut from the turbocharger rotor is presented in Figure 8b.

**Figure 9:**
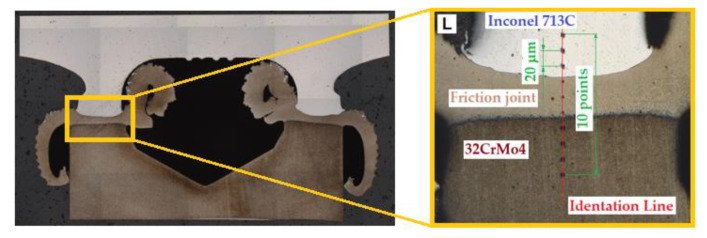
Scheme for measuring the microhardness of a welded joint.

**Figure 10:**
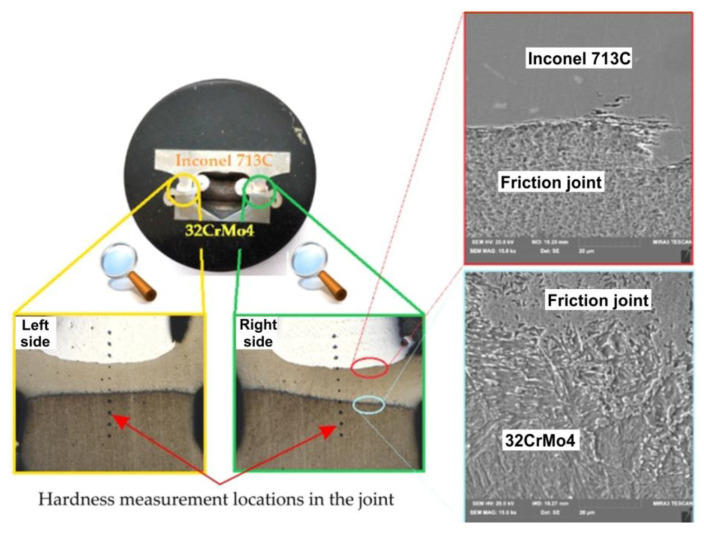
The microstructure of a friction-welded joint (32CrMo4 + Inconel 713C) in two characteristic places: on the 32CrMo4 steel side and the Inconel 713C side.

After the cut samples were positioned in epoxy resin (Figure 7c), each sample was polished using discs with a grain size of 120 to 2500 and then polished with a diamond paste (grain size 3 μm and 1 μm). In order to reveal the microstructure, the samples were etched with the Mi-19 Fe reagent (chemical composition: 3 g FeCl_3_, 20 g HCl, 90 mL C_2_H_5_OH). The joint hardness test was carried out on the samples before the etching stage. Figure 9 shows a diagram of the measurement points for the microhardness of the friction-welded joints. For each joint, the hardness was measured at ten points. Three measurement points were located in Inconel 713C, three points in the obtained weld, and the remaining four in the 32CrMo4 material. The test sample and the locations where the joint microstructure was analysed are shown in Figure 10.

## 3. Results

### 3.1. Hardness of the Joints

**Figure 11:**
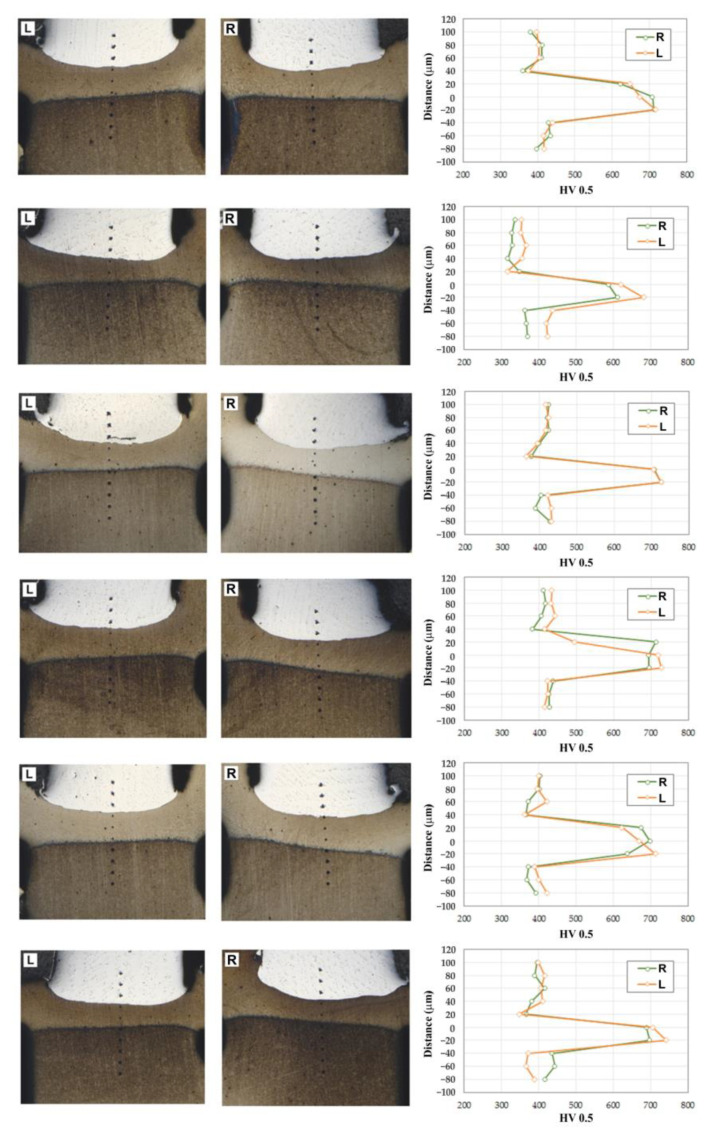
Microhardness of the 32CrMo4-Inconel 713C RFWed joint (L—left_side, R—right side).

The average values of hardness for the BM were found to be 420 HV0.5 for Inconel 713C and 360 HV0.5 for 32CrMo4 steel. The hardness values obtained, as shown in Figure 10, were carried out at 0.2 mm intervals. Differences in hardness in the heat-affected zone and base metal were also observed. The hardness distribution of the specimens is presented in Figure 11. It can be seen that the hardness decreases in the thermo-mechanically affected zone from Inconel 713C to 32CrMo4. The hardness of the partially deformed steel zone increases with increasing friction pressure.

Increasing the friction pressure causes martensite formation and thus increases the hardness in the joint zone. The interface of the weld is intensively mixed, and a strong bonding line between joined materials is formed, as also observed by Kong et al. [50]. After RFW, the welds have three zones: the thermo-mechanically affected zone, the heat-affected zone, and the BM zone. In both the heat-affected zone and the thermo-mechanically affected zone, an increase in the hardness of the material was observed compared with the base material. According to the investigations of friction welding of dissimilar materials by Kong et al. [50], the materials experienced work hardening, which results in changes in their mechanical properties. Due to the different work hardening of welded alloys, these properties contribute to the variation in hardness across the weld.

### 3.2. Tensile Strength

The results of the ultimate tensile strength R_m_ obtained for the joints produced with various values of friction welding parameters are presented in Table 4. The tensile strength decreases with the increasing rotational speed of the shaft (Table 4). Using higher rotational speeds increases the amount of heat generated. Too high a rotational speed may result in local melting of the joined materials. It can also lead to a loss of uniformity in the joint, affect the microstructure of the joint, and result in the formation of precipitations that weaken the welded elements.

**Figure 12:**
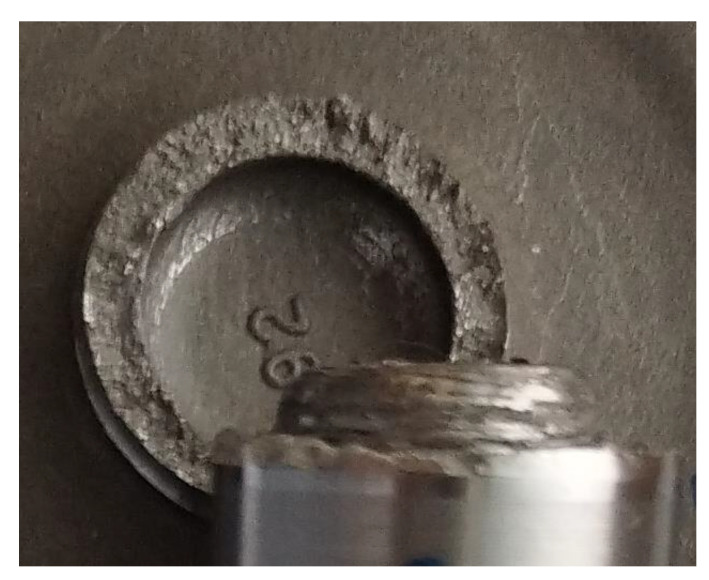
View of the sample fractured from the weld zone.

All experiments were performed in triplicate for each parameter. It has been shown that increasing the frictional pressure reduces the tensile strength, even if the change is not significant. The tensile strength of the weld area in all analysed variants is higher than the tensile strength of the base metal (32CrMo4 steel). In the tensile strength tests, the rupture of the welded joint always occurred on the 32CrMo4 steel side (Figure 12). The tensile strength of the joints decreases with increasing friction pressure.

In order to determine the effect of the friction pressure on the tensile strength of the weld, the friction pressure was applied at three levels: 21, 24, and 27 MPa. The tensile strength values obtained are shown in Table 4. The highest tensile strength of 1222 MPa is achieved at a friction pressure of 21 MPa. As the frictional pressure increases to 24 and 27 MPa, the tensile strength values are approximately 1100 MPa and 1050 MPa, respectively. This shows that increasing the frictional pressure reduces the tensile strength, even if the change in pressure is not significant.

As already mentioned, all the welded specimens in the tensile tests failed on the 32CrMo4 steel side. The strength of the weld area in all the welds turned out to be higher than the tensile strength of the 32CrMo4 material. The occurrence of a ductile fracture was observed in the 32CrMo4 material. During a ductile fracture, the formation and joining of cracks take place due to the plastic flow of the material. Ductile fracture occurs by void nucleation and growth and usually starts with particles of a different phase. The distribution of particles in the joint was relatively uniform, so cracking was initiated by the grains. Such cracking is called transcrystalline. The incompatibility of strains between the hard particles and the matrix resulted in the generation of dislocations in the matrix during deformation. Because the ductile matrix contains brittle particles of a different phase, such particles are not able to accommodate large plastic deformations of the matrix; therefore, even when the plastic deformations of the matrix are not large, the stress from the dislocation reaches a value sufficient to cause cracking of the particles or, if the boundary of particle-matrix is weak, decohesion at the interface. Both types of behaviour result in the formation of microvoids on the particles. During further deformation, local necks are formed between the microvoids in the material, and when these necks break, the voids join together. Since the processes occurring during ductile fracture are usually associated with particles from another phase, it is not surprising that ductility strongly depends on the size, density, and plastic deformation capacity of the particles from another phase and the strength of the particle-matrix interface. As a result of the thermomechanical stresses occurring during friction welding in the fully deformed zone (the thermomechanical impact zone) and when the temperature exceeds 650 °C, the content of the γ phase in the γ′ austenitic solution decreases as a result of the diffusion effect. As a result, the value of the tensile strength of the welded joint decreases. The occurrence of brittle cracks at a very low percentage of elongation was observed in the welding tests carried out. No significant changes in hardness were observed in the welding process for the tested parameters.

**Figure 13:**
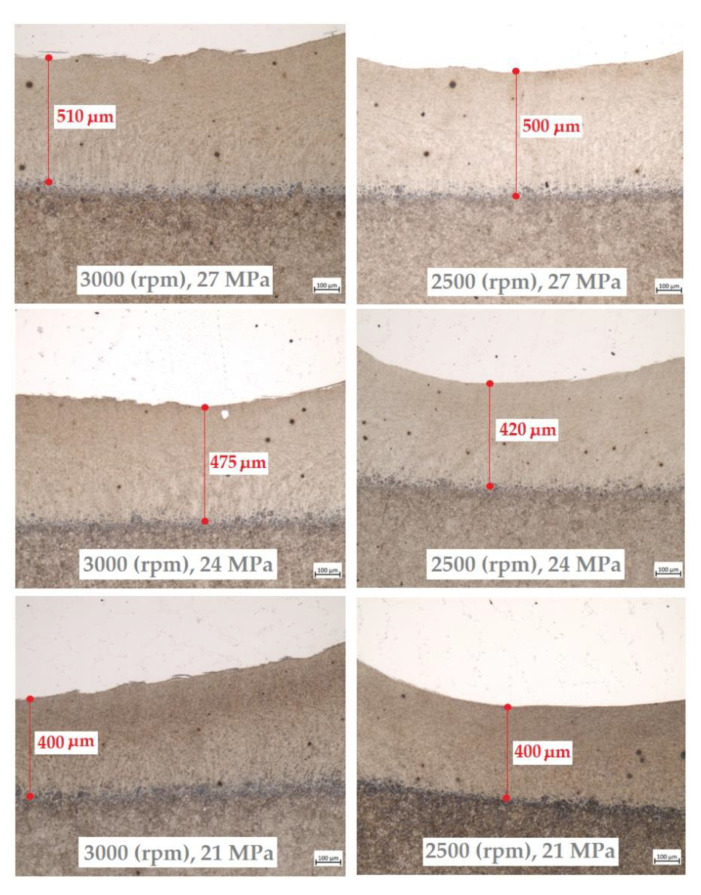
Results of the thickness of the resulting weld.

Figure 13 shows the dimensions of the weld resulting from the thermal welding process. The measurement was made at the point where the weld was the thinnest. The measurements clearly show that weld thickness is closely related to the value of the friction pressure. The thickness of the weld increases with the increase in friction pressure. This is also directly transferred in the same way to the resulting flash thickness.

### 3.3. Optical Microscopy

A wide group of nickel alloys are precipitation strengthening alloys, often called nickel superalloys. Superalloys are generally referred to as alloys that can work at high temperatures and in an environment of oxidising gases. These alloys are characterised by very high strength and corrosion resistance at elevated temperatures. Nickel matrix superalloys have a very complex chemical composition—a typical alloy contains about 15 elements. Cobalt, iron, chromium, molybdenum, tungsten, and vanadium promote the formation of an austenitic matrix of nickel superalloys. First of all, aluminium, but also niobium, tantalum and titanium, favour the formation of a reinforcing phase, called γ′ in superalloys. Chromium, molybdenum, tungsten, vanadium, niobium, tantalum, and titanium are carbide-forming elements in superalloys. In addition, chromium, aluminium, and tantalum improve the oxidation resistance of superalloys. The introduction of new methods of producing nickel superalloys was accompanied by the evolution of the microstructure. The following phases can be observed in nickel superalloys:

- austenitic disordered matrix γ,
- ordered γ′ phase coherent with the matrix,
- MC, M_23_C_6_ and M_6_C carbides.

**Figure 14:**
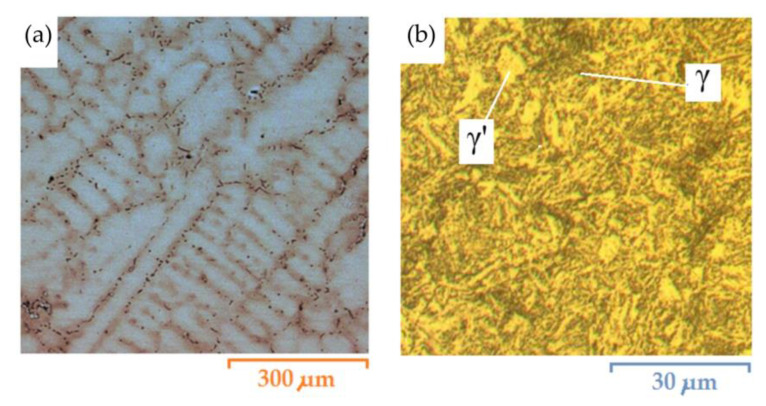
Microstructure of: (**a**) Inconel 713C and (**b**) 32CrMo4 steel.

The microscopic images of the 32CrMo4 steel material are shown in Figure 14b, and those of the nickel-based superalloy Inconel 713C in Figure 14a. It can be observed that the microstructure of 32CrMo4 steel has a ferritic (yellow colour) and pearlitic (dark colour) structure. The Inconel 713C microstructure is dendritic, as shown in Figure 14a. Moreover, the grain shows chain formations of carbides at the grain boundaries. The Inconel 713C alloy is characterised by a dendritic microstructure that consists of a γ′-Ni_3_ phase (Al, Ti) and nickel austenite grains with cobalt, iron, chromium, and molybdenium.

The microstructure of the Inconel 713C superalloy consists of grain boundary type carbides. These carbides are formed by carbide-forming elements such as chromium, tungsten, and molybdenum. These carbides are already visible in the structure under the optical microscope. Ni-Cr-Al-based superalloys are precipitation hardening alloys with γ′ phase reinforcement. High temperature reduces the mechanical strength and corrosion resistance of the Inconel 713C alloy due to the effect of high temperature on the degradation of the primary elements. Harmful M_23_C_6_ and M_7_C_3_ carbides found at the grain boundaries weaken grain boundaries. The high precipitation hardening temperature causes the formation of spherical γ′ precipitates.

**Figure 15:**
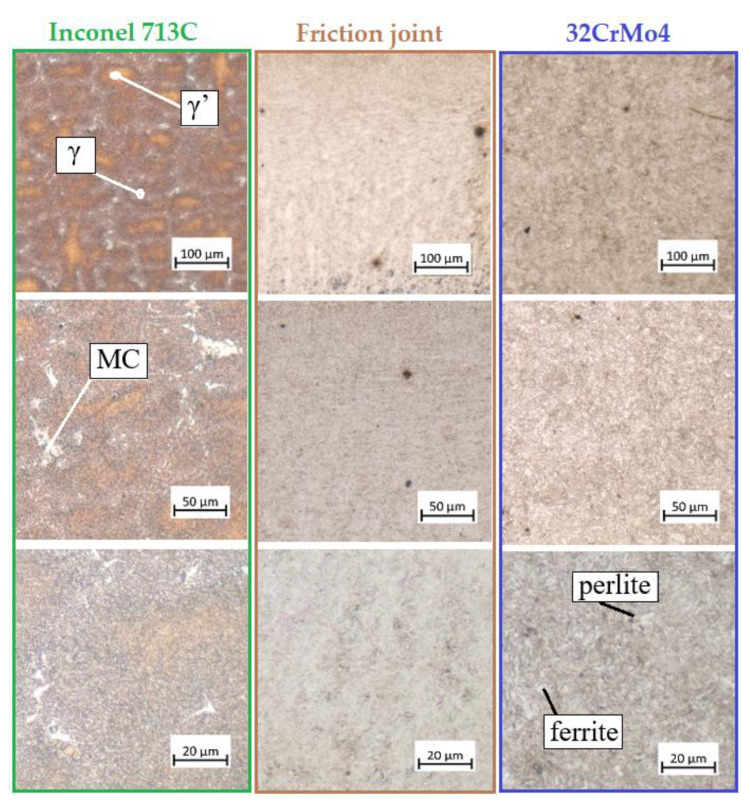
The microstructure of the welded joint area.

Figure 15 shows the microstructure of three areas of the welded joint at different magnifications. The structure of Inconel 713C is marked in green. The microstructure of the Inconel 713C alloy is dendritic and consists of a solid γ phase (grey) and a γ′ phase (brown). In addition, chain formations of MC carbides can be seen at the grain boundaries. The microstructure of 32CrMo4 steel is marked in blue. It has a ferritic (light colour) and pearlitic (dark colour) structure. The microstructure of the resulting joint (weld) is marked in brown and has a characteristic spongy structure. The structure of the weld is a result of mixing 32CrMo4 and Inconel 713C materials.

### 3.4. Scanning Electron Microscopy

Macroscopic examination of the welded specimens showed that all the specimens were shortened in the direction of the applied load. The more the material was plasticised by the high temperature generated in the joint, the greater the shortening. Due to the better plastic properties of 32CrMo4 steel, shortening of the joint was observed on the side of this material. This is due to the fact that 32CrMo4 steel loses hardness at high temperatures and is more susceptible to plastic deformation than the material of the turbocharger rotor (Inconel 713C). As a result, Inconel 713C superalloys showed no distortion. Materials with less strength in dissimilar friction stir welding tend to plastically deform under high temperatures and dynamic force [44,51,52]. The structure of the γ-phase of the nickel-based superalloy is an austenitic body-centred cubic lattice that contains a large number of elements, such as Cr, Mo, W, and Co.

**Figure 16:**
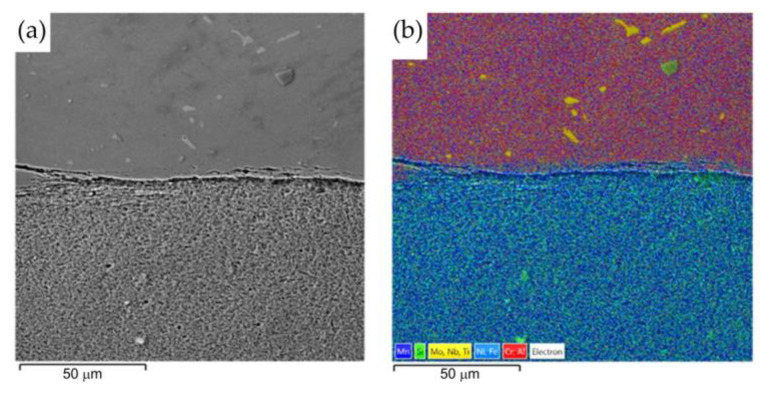
Friction-welded joint: (**a**) SEM microstructure and (**b**) surface distribution of elements at the Inconel 713C–weld interface.

**Figure 17:**
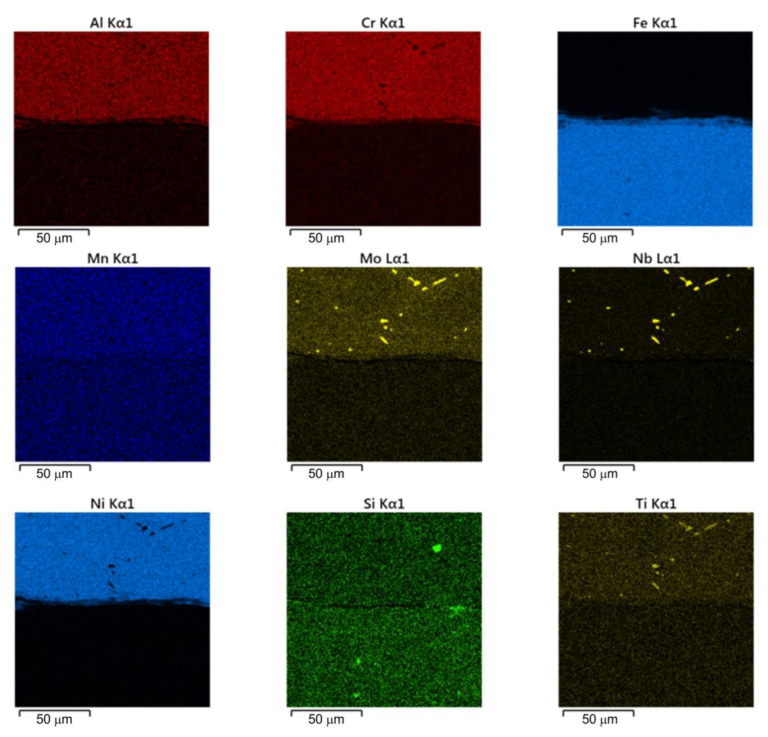
Surface distribution of the elements in a RFWed joint at the Inconel 713C-weld interface.

Figure 16 shows a welded joint from the Inconel 713C material side. Analysis of the microstructure of Inconel 713C shows a matrix consisting of the γ phase characterised by uniform distribution during crystallisation of carbide at the interface of the weld with the base material. Figure 16a shows a continuous layer of secondary carbides arranged in parallel to the welded joint. Figure 16b shows the mapping analysis of the resulting welded joint and the formation of secondary carbides in the form of the above-mentioned continuous layer. The results of the analysis clearly show that elements such as niobium, molybdenium, titanium, and chromium support the formation of the structure of the secondary carbides. Detailed surface distributions of the elements in the analysed areas (from the Inconel 713C side) are shown in Figure 17.

**Figure 18:**
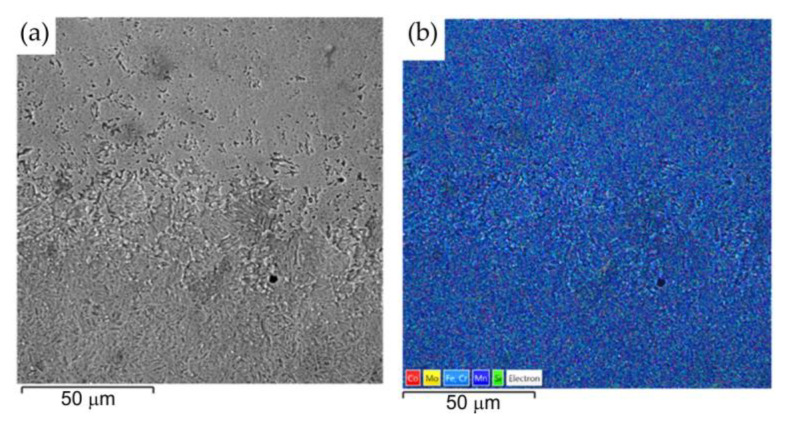
Friction-welded joint: (**a**) SEM microstructure and (**b**) surface distribution of elements at the 32CrMo4–weld interface.

Figure 18 shows a welded joint from the 32CrMo4 steel side. The analysis of the microstructure of the joint shows a uniformly distributed microstructure characterised by a uniform distribution of alloying elements of the base material (32CrMo4 steel). However, the microstructure of the weld flash on the 32CrMo4 side consists of fine and equiaxed grains compared with the base metal [50]. Differences in the microstructure of the 32CrMo4 material after the friction welding process were also observed. Microstructures formed as a result of the hardening of the steel. This occurs in the form of martensite. The visible martensite crystals are fine. The microstructure of the steel in the thermo-mechanically affected zone and the heat-affected zone is acicular and does not show the presence of dendrites.

**Figure 19:**
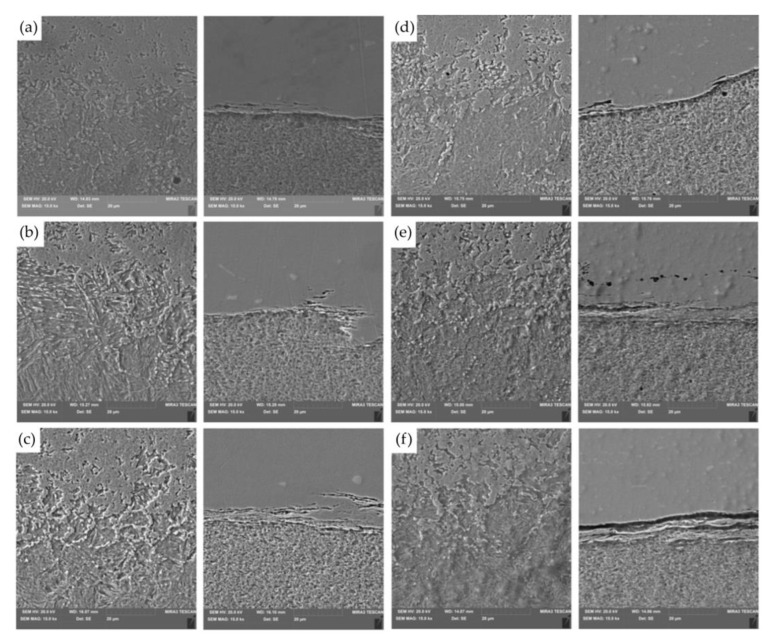
Friction welded joint: (**a**) the microstructure of joint A; (**b**) the microstructure of joint B; (**c**) the microstructure of joint C; (**d**) the microstructure of joint D; (**e**) the microstructure of joint E; and (**f**) the microstructure of joint F.

Studies show a significant contribution of niobium in the dark, uniformly coloured, irregular formations of the transition zone (Figure 19). Analysis of the microstructure of Inconel 713C shows a matrix consisting of the γ phase characterised by uniform distribution during crystallisation of carbide at the interface of the weld with the 32CrMo4 material. The second phase is Fe-rich. During the RFW process, the phenomenon of diffusion across interfacial boundaries in the solid state occurs. The morphology of the joint is the result of the mechanical mixing of the refined grains of both materials.

For RFWed joints, it is most desirable that the HAZ has an even distribution (same width) along the resultant friction weld. The HAZ zone, where the temperature was lower, is characterised by strongly bent bands in the radial direction. For example, in Figure 19b, the difference in the topography of the structure near the welded joint can be seen. The friction welding process did not cause a loss of the structure’s banding but a change in the arrangement of the bands from vertical and diagonal to horizontal (radial). In addition, the horizontal bands are disturbed by lines that are most likely grain boundaries. The coniferous character of the base material was completely lost in the weld zone. In the friction-welded joints, it is not possible to isolate the interface. The weld shows a homogeneous, spongy microstructure. Regardless of the nature of changes in the observed microstructure, the heat generated in the RFW process resulted in softening of the material. In the HAZ zone, the hardness decreased on average by approximately 80 HV0.5. However, the HAZ zone has a more homogeneous microstructure than the base material.

Figure 19b shows a martensitic structure (in the form of characteristic needles). It was created as a result of the thermomechanical impact of the joined surfaces (32CrMo4 steel side) during the welding process. As a result of the high temperature and relatively quick cooling time, martensite nuclei in the form of characteristic needles were separated in the primary ferritic-pearlitic structure. In the HAZ, the temperature is between A1 and A3, resulting in partial transformation to austenite, which is transformed to perlite and ferrite during cooling. Heating of the steel above A3 and cooling leads to refinement of the structure as a result of austenite transformation to ferrite and perlite [50].

## 4. Conclusions

In this article, the microstructure, hardness, and tensile strength of rotary friction welded Inconel 713C-32CrMo4 steel joints were studied, with particular emphasis on changes in the joint microstructure and its mechanical properties. Based on the results of the research, the main conclusions are:

- It is possible to conduct solid-state friction welding of an Inconel 713C and 32CrMo4 steel material pair.
- Tensile tests of the RFWed joints showed that the combination of 32CrMo4 alloy steel and Inconel 713C fully meets the minimum values assumed in the automotive industry (R_m_ > 760 MPa).
- The tensile strength of an RFWed joint decreases with increasing friction pressure. The fracture of the joint always occurs on the side of the 32CrMo4 material.
- An increase in the hardness value in the thermo-mechanically affected zone for the 32CrMo4 steel side was observed with increasing friction pressure.
- The highest tensile strength was obtained at a level of 1222 MPa in the friction welding experiments of sample no A (rotational speed of shaft 2500 rpm, friction pressure 21 MPa).
- SEM analysis showed carbide deposition at the grain boundaries in the thermo-mechanically affected zone on the Inconel 713C side.
- No influence of the rotational speed (in the tested range) in the friction process on the mechanical properties and microstructure of the joint was observed.
- The friction weld microstructure consisted of fine-grained, equiaxed grains formed as a result of dynamic recrystallisation. In addition, the flash was formed on the 32CrMo4 steel side.

## Figures and Tables

**Table 1:** Chemical composition of 32CrMo4 material (wt.%).

C	Si	Mn	P	S	Cr	Mo	Cu
0.41	0.35	0.85	0.015	0.025	1.15	0.25	0.15

**Table 2:** Chemical composition of Inconel 713C (wt.%).

Ni	Cr	Al	Mo	Nb	Zr	W	Cu	Fe
70.30	13.20	5.8	4.4	2.1	0.04	0.30	0.47	0.15

**Table 3:** Friction welding parameters.

Sample	Rotational Speed of Shaft (rpm)	Friction Pressure (MPa)	Friction Time (s)
A	2500	21	4
B	3000	21	4
C	2500	24	4
D	3000	24	4
E	2500	27	4
F	3000	27	4

**Table 4:** Ultimate tensile strength R_m_ of the RFWed joint.

Rotational Speed of Shaft (rpm)	Friction Pressure (MPa)	Tensile Strength R_m_ (MPa)
2500	21	1222
3000	21	1207
2500	24	1106
3000	24	1095
2500	27	1057
3000	27	1028

## Data Availability

The data presented in this study are available on request from the corresponding author.
